# Radiofrequency Ablation: A Minimally Invasive Approach in Kidney Tumor Management

**DOI:** 10.3390/cancers2041895

**Published:** 2010-11-17

**Authors:** Maciej Salagierski, Marek S. Salagierski

**Affiliations:** 1 I Urology Department, Medical University of Lodz, Poland; 2 II Urology Department, Medical University of Lodz, Poland; E-Mail: msalagierski@gazeta.pl

**Keywords:** minimally invasive technique, renal tumor, radiofrequency ablation

## Abstract

The management and diagnosis of renal tumors have changed significantly over the last decade. Due to advances in imaging techniques, more than 50% of kidney tumors are discovered incidentally and many of them represent an early stage lesion. This has stimulated the development of nephron-sparing surgery and of the minimally invasive treatment options including ablative techniques, *i.e.*, radiofrequency ablation (RFA) and cryoablation. The objective of the minimally invasive approach is to preserve the renal function and to lower the perioperative morbidity. RFA involves inducing the coagulative necrosis of tumor tissue. Being probably one of the least invasive procedures in kidney tumor management, RFA may be performed percutaneously under ultrasound (US), computed tomography (CT) or magnetic resonance (MR) guidance. Most of the studies show that the RFA procedure is efficient, safe and has a low complication rate. Due to the still limited data on the oncological outcome of RFA, the indication for this intervention remains limited to selected patients with small organ-confined renal tumors and contraindication to surgery or who have a solitary kidney. The aim of our study is to review the literature on RFA of kidney tumors.

## 1. Introduction

At present, most of the detected kidney tumors constitute incidentally detected small renal masses (SRM). These small lesions possess a relatively slow clinical progression with less than 10% of tumors leading to metastases or death [1]. SRM have been also reported to have a very slow growth rate of approximately 0.1–0.4 cm a year [2]. Unfortunately, with the current status of knowledge, the natural history of SRM remains difficult to predict and an aggressive tumor phenotype is not uncommon [3]. Importantly, it has been shown that a less invasive approach in small kidney tumors management does not affect cancer specific survival [4]. In addition, the number of patients with renal insufficiency has been steadily growing. In this setting, the preservation of renal parenchyma and function is non-negligible. As a consequence, treatment of SRM has evolved greatly in the last years with a constant trend for less invasive and nephron-sparing approaches. Currently, nephron-sparing surgery, *i.e.* partial nephrectomy, is recommended for the management of small renal tumors (<4 cm; T1a). Similarly, there is a growing interest in minimally invasive therapeutic strategies, e.g., ablative techniques, which additionally do not lead to the loss of nephrons secondary to the clamping of the renal pedicle [5]. Although the use of radiofrequency energy is still limited to surgically unfit patients, its application appears safe and leads to the reduction in morbidity and costs [6].

## 2. Limitations and Technical Improvements

Percutaneous radiofrequency ablation (pRFA) has demonstrated encouraging results as a minimally invasive and safe technique for the treatment of kidney tumors. The procedure has already been performed in many oncology centers and the reported treatment results were mostly very satisfying [7,8,9,10,11]. Promising mid-term results were also reported where pRFA was used to treat tumors in a solitary kidney [12,13].

Technically, RFA involves inducing the coagulative necrosis of tumor tissue via needle electrodes [14]. The ultimate goal of RFA is to completely destroy tumor cells. The procedure can be performed using one or more RF probes, depending on the tumor size. Importantly, by using at least two conducting probes it becomes feasible to obtain the ‘overlapping effect’ of RF-induced tissue coagulation. It is thought that this phenomenon occurs when the probes are within approximately 1–2 cm of each other [15]. It has been suggested that the ‘overlapping effect’ results in a more effective tissue ablation.

Actually, the monitoring of the ablation process constitutes the main concern in regard to pRFA. Unlike in cryoablation, where iceball formation can be monitored by US, CT or MRI, during pRFA it is difficult to follow or delineate the developing thermal lesion. It is not only impossible to observe RF-induced changes occurring in tissue with increasing temperature, but also tissue vaporization and the formation of steam bubbles restrict visibility. Most probably due to the monitoring of the ablation zone, cryoablation is associated with a significantly lower risk of local progression than RFA and results in better oncologic outcomes [16]. Therefore, real-time monitoring appears necessary to precisely and completely coagulate a tumor, *i.e*., to achieve a temperature of at least 70 °C (regarded as necessary for an effective ablation) not only inside a tumor but also at its borders. Carey *et al*. applied the especially designed RFA equipment, *i.e.*, Luxtron data module (Luxtron Corporation, Santa Clara, CA) consisting of two fiberoptic non-conducting temperature RF probes accompanied by the ‘traditional’ RF electrode [11]. The fiberoptic probes were used to monitor the temperature at the tumor margin. According to Carey and his colleagues, the majority of renal masses between 3 and 5 cm can be treated with a single pRFA session [11]. They reported a complete tissue necrosis confirmed by nicotinamide adenine dinucleotide (NADH) viability staining in 100% of patients after the initial RFA session. The demonstrated pathologically proved, excellent short-term results might be related to the precise RF energy distribution. Similarly, in one recent studies, traditional RF probes were used as non-active thermal probes to independently monitor the temperature within the tumor during the US-guided intervention [17]. Forty-two larger renal lesions ranging from 3 to 6 cm were treated with pRFA. In 14 patients (33%), RF energy was used to coagulate tumors exceeding 4 cm. Thirty-eight tumors (90%) showed no contrast enhancement on CT. Four incompletely ablated tumors (10%) were successfully treated with the second ablation session. Although two major and two minor complications occurred in this series, the procedure appears safe and efficient. Certainly, monitoring of the temperature inside the tumor leads to a better control of percutaneous procedure by providing reliable information about the ablation process. Therefore, temperature monitoring might importantly improve the oncological outcome of RFA procedure. According to the authors and in agreement with the study of Carey *et al*., the real time monitored intervention could be recommended for surgically unfit patients with up to 6 cm organ-confined kidney masses. Nevertheless, with the current status of knowledge, the application of minimally invasive techniques for treating larger kidney tumors ranging from 4 to 7 cm (T1b) remains controversial. Further studies and longer follow-ups are necessary to better evaluate safety and oncological outcome of RFA [18].

## 3. Cost-effectiveness and Complications

Minimally invasive techniques generally lead to cost reduction related to decreased morbidity, quick recovery and shorter hospital stay. The procedure can be performed under local anesthesia with conscious sedation. Pandharipande *et al.* described a much increased pRFA cost-effectiveness compared to ‘traditional’ nephron-sparing surgery [6]. Although in most centers patients are usually released from hospital one day after intervention, pRFA of kidney tumors might be even performed in an outpatient setting. The most common complications of RFA include pain and paresthesis reported in less than 5% of individuals. Other observed complications included: perinephric hematomas, neuralgia, transient hematuria, ureteropelvic junction obstruction, urine leak, bowel injury, massive bleeding, pneumothorax and liver burns [19]. Carrafiello *et al.* recently described the ‘post RFA syndrome’ consisting of unspecific symptoms including fever, nausea, vomiting and/or malaise. According to the authors, the post-ablation syndrome is a common phenomenon after RFA of solid tumors occurring in approximately one-third of treated patients [20]. Other studies, including our own experience [21], do not corroborate such a high prevalence of ‘post RFA syndrome’. The fairly frequently reported urine leak occurs as a consequence of the thermal injury of the renal excretory system, which could happen when the RFA probe is inserted in proximity of the calyces. In our own series we observed urine leak in three patients who underwent RF-assisted partial nephrectomy. RF energy was used to perform partial nephrectomy without clamping renal pedicle [22]. Fortunately, in most cases urine leak resolves spontaneously, sometimes it requires endoscopic management. It is believed that the complication rate may significantly decrease by stringent patient selection based on patient and tumor characteristics.

## 4. Follow-up

Follow-up after renal ablative therapy remains troublesome. There is no perfect tool for detecting local progression. The absence of contrast enhancement on CT does not exclude the presence of viable cancer cells. The results of some studies using NADH staining, contrary to the findings of Carey and his colleagues [11] (see above), showed the absence of total tumor necrosis and presence of cancer cells in un-enhancing renal masses [23,24]. However, the application of NADH staining by itself to assess the presence of viable cancers cells has been questioned. In particular, a positive staining just after intervention might be not accurate enough in assessing cell processes and wrongly interpreted as local progression. According to Anderson *et al.* [25], longer post RFA time of NADH inactivation should be taken into account for the proper determination of cellular viability. The animal study revealed scarce regions within the ablation zone with the disappearance of NADH staining as late as 150 minutes after the ablation session. This residual staining might be explained by slower enzymatic changes following RFA in some cases. Furthermore, some authors suggest the residual contrast enhancement after RFA might be not only related to the residual tumor but also to inflammation in the area surrounding the ablation zone [26]. Unfortunately, the reported mean follow-up after ablation of kidney tumors is generally short and does not exceed three years in most of the large RFA series.

## 5. RFA and Histopathology

Approximately 20% of small renal tumors are considered to be benign [2] and probably do not require any treatment. Therefore, in many centers, percutaneous biopsy is performed before or in conjunction with the ablation procedure. Despite the fact that overall accuracy of renal biopsy is around 80% [27], it is advocated to biopsy renal tumors prior to RFA. The underuse of renal tumor biopsy and lack of biopsy-proven material constitute a major limitation of several RFA series. Biopsy result might be of great importance especially for patients with disease progression who will be exposed to antiangiogenic therapy. Because the ablated renal tumor is left *in situ*, it is not available for complete pathological evaluation. Hence, definitive histopathological confirmation about the diagnosis, margins and completeness of tumor cell killing cannot be obtained after intervention. The clinical significance of post-ablation biopsy also remains unsettled and it is a matter of debate whether to perform a routine post-ablation biopsy to assess the RF-induced tumor tissue necrosis.

## 6. Conclusions

Taken together, percutaneous RFA is a minimally invasive technique with generally low complication rates, which seems to represent an excellent therapeutic modality for patients with small exophytic renal tumors and contraindication to surgery. With the implementation of reliable real-time monitoring devices e.g., fiberoptic probes, the RFA procedure is highly efficient and oncological outcome appears to improve. Nevertheless, further studies and longer follow-ups are necessary to evaluate safety and oncological outcome of this technique.
